# Diagnostic agreement between three point-of-care glucose and β-hydroxybutyrate meters and reference laboratory methods in stingrays

**DOI:** 10.3389/fvets.2023.1254340

**Published:** 2023-12-20

**Authors:** Nicholas G. Dannemiller, Carolyn Cray, Lori S. Westmoreland, Emily F. Christiansen

**Affiliations:** ^1^Department of Clinical Sciences, College of Veterinary Medicine, North Carolina State University, Raleigh, NC, United States; ^2^North Carolina Aquariums, Raleigh, NC, United States; ^3^Division of Comparative Pathology, Department of Pathology and Laboratory Medicine, University of Miami Miller School of Medicine, Miami, FL, United States

**Keywords:** batoid, clinical pathology, elasmobranch, ketone, metabolism

## Abstract

Point-of-care (POC) glucose and β-hydroxybutyrate (β-HB) meters can potentially provide rapid insight into an elasmobranch’s metabolic state in clinical and field research settings. This study evaluated the diagnostic agreement of three commercial POC meters against reference laboratory methods for glucose and β-HB concentrations in stingrays. Blood was collected during anesthetized exams from 28 stingrays representing four species: cownose rays (*Rhinoptera bonasus*), Atlantic stingrays (*Hypanus sabina*), southern stingrays (*Hypanus americanus*), and yellow stingrays (*Urobatis jamaicensis*). Glucose and β-HB concentrations were measured with each POC meter using whole blood and plasma; in parallel, plasma glucose and β-HB concentrations were measured via reference laboratory methods. Agreement between POC meters and reference laboratory methods was assessed using Bland–Altman methods, Passing-Bablok regression, observed total error, percent relative error, and linear mixed effect models. Plasma glucose and β-HB concentrations determined by reference laboratory methods ranged from <20–63 mg/dL to 0.05–5.38 mmol/L, respectively. One human POC meter—the Precision Xtra—showed the greatest agreement with reference laboratory methods when measuring glucose with whole blood [mean bias and 95% CI: 0 (−3–4) mg/dL] and β-HB with plasma [mean bias and 95% CI: 0.1 (−0.04–0.2) mmol/L]. Stingray sex, weight, buffy coat, and packed cell volume did not significantly affect the agreement between POC meters and reference laboratory methods. Across all three POC meters, mean bias and imprecision for plasma β-HB concentrations were relatively small (0–0.1 mmol/L and 0%, respectively). Utilizing POC meters to measure glucose and β-HB in stingrays may be viable when reference methods are unavailable.

## 1 Introduction

Elasmobranchs (sharks, skates, and rays) are an ancient and diverse group of cartilaginous fish that play essential ecologic roles in aquatic ecosystems globally. Over 200 stingray species are grouped in the order Myliobatiformes, suborder Myliobatoidei (1). Most stingrays are demersal and can be found in tropical to subtropical marine or freshwater habitats. Stingray species are increasingly listed as vulnerable or endangered on the International Union for Conservation of Nature Red List of Threatened Species, an unfortunate trend shared by all elasmobranchs, with almost 1/3 being threatened with extinction (2). Consequently, stingrays are common in public aquariums due to their ecological importance and increasing need for conservation.

One unique physiologic feature of elasmobranchs is their preferential use of ketone bodies—especially β-hydroxybutyrate (β-HB)—rather than fatty acids for aerobic metabolism under normal conditions, predominately in skeletal and cardiac muscle (3–5). Besides providing buoyancy, elasmobranchs’ large, lipid-dense livers serve as ketogenic powerhouses (4). Within the liver, acetyl coenzyme A is formed from long-chain fatty acids by mitochondrial β-oxidation and utilized in numerous metabolic reactions, including generating ketone bodies. Ketone bodies are highly water-soluble, energy-rich fuels exported to extra-hepatic tissues without the need for protein-binding (3). Furthermore, ketone bodies likely play a significant role in supporting metabolism during periods of fasting in elasmobranchs (3–6). While the concentration of ketone bodies in fed elasmobranchs is similar to that observed in fasted (0–3 mmol/L) or ketoacidotic (> 3 mmol/L) mammals, marked ketosis has been found during starvation or prolonged fasting (4).

Glucose is potentially of less metabolic importance in elasmobranchs, although differences between tissues, species, and metabolic states exist (3–5, 7, 8). Plasma glucose concentrations are often low relative to teleosts and terrestrial vertebrates and appear relatively unchanged after feeding or during long-term fasting (3). Some elasmobranch species reportedly tolerate wide ranges of plasma glucose concentrations with no observed clinical effects (3). Nevertheless, a stress-induced increase in catecholamines or corticosteroids (e.g., 1α-hydroxycorticosterone) has been speculated to possibly correlate with increased glucose mobilization, which may reflect increased demand as an oxidative fuel source or supply increased rates of anaerobic glycolysis (3–5, 9, 10). This is best highlighted by the hyperglycemia observed in elasmobranchs as a response to various stressors, including capture, handling, and transport (10–13).

Conventional measurement of glucose and β-HB concentrations in veterinary medicine requires serum or plasma to be submitted to a reference laboratory. As an alternative to laboratory-based assays in human and veterinary medicine, point-of-care (POC) meters typically provide rapid results, require smaller sample volumes, and require no centrifugation or cold chain before testing (14). Within veterinary medicine, the diagnostic accuracy of POC glucose and β-HB meters have been explored in multiple domestic (15–21) and non-domestic species (22–28). Point-of-care glucose and β-HB meters may aid veterinarians, researchers, and wildlife managers working with elasmobranchs, including stingrays, by providing almost instant insight into their physiologic stress or metabolic state.

This study evaluated the diagnostic agreement of three commercial POC glucose and β-HB meters with reference laboratory methods for glucose and β-HB concentrations in stingrays. We hypothesized that the results from the POC meters evaluated would have sufficient agreement with reference laboratory methods and precision for clinical and field research purposes for glucose and β-HB concentrations in stingrays.

## 2 Materials and methods

### 2.1 POC meters and reference laboratory

Glucose and β-HB concentrations determined by three commercially available POC meters were compared to results from reference laboratory methods. Each meter measures glucose or β-HB concentrations independently using corresponding test strips. All three meters measure glucose concentration via a glucose-1-dehydrogenase enzymatic reaction in which glucose is converted to gluconolactone using a coenzyme to convert nicotinamide adenine dinucleotide (NAD+) to its reduced form NADH. NADH is re-oxidized to NAD+ by a redox mediator, which is measured via amperometry (i.e., an electrical current) and is directly proportional to glucose concentration (14). Similarly, all three meters measure β-HB concentration via a β-HB-dehydrogenase enzymatic reaction in which β-HB is converted to acetoacetate, leading to a concurrent equimolar reduction of NAD+ to NADH. NADH is re-oxidized to NAD+ by a redox mediator, which is measured via amperometry (i.e., an electrical current) and is directly proportional to β-HB concentration (14). Each meter and its respective test strips were stored and operated following manufacturer instructions; only the correct corresponding test strips provided by the manufacturer were used for each respective meter. Before all data collection, meters were calibrated following manufacturer instructions. None of the meters were operated outside their recommended temperature (10–50°C or 50–122°F) and humidity (10–90%) ranges.

The first POC meter evaluated was the BHBCheck Plus Blood Ketone & Glucose Monitoring System (PortaCheck, Moorestown, NJ, United States), a veterinary-licensed POC meter that requires 0.7 μL of whole blood, measures glucose concentrations within 20–600 mg/dL, and β-HB concentrations within 0.1–8.0 mmol/L. The BHBCheck Plus meter is intended for heparinized or non-heparinized bovine venous whole blood with a 20–40% packed cell volume (PCV).

The second POC meter evaluated was the Precision Xtra Blood Glucose & Ketone Monitoring System (Abbott Laboratories, Chicago, IL, United States), a human-licensed POC meter that requires 1.5 μL of whole blood, measures glucose concentrations within 20–500 mg/dL, and β-HB concentrations within 0–8.0 mmol/L. The Precision Xtra meter is intended for freshly drawn human capillary whole blood with a 30–60% PCV.

The third POC meter evaluated was the Nova Vet™ Ketone/Glucose Meter (Nova Biomedical, Waltham, MA, United States), a veterinary-licensed POC meter that requires 0.4 μL whole blood for glucose and 0.8 μL whole blood for β-HB, measures glucose concentrations within 20–600 mg/dL, and β-HB concentrations within 0.1–8.0 mmol/L. The Nova Vet™ meter is intended for heparinized or non-heparinized bovine whole blood with a 25–60% PCV.

Plasma glucose and β-HB concentrations were measured using a VITROS® 5600 analyzer (Ortho Clinical Diagnostics, Rochester, NY, United States) maintained at the reference laboratory (University of Miami, Miami, FL, United States). The analyzer is maintained per manufacturer instructions and quality assurance best practices. Glucose was determined by a colorimetric glucose oxidase assay based on peroxidase-catalyzed oxidative coupling, producing a red-colored quinoneimine dye with color intensity directly proportional to glucose concentration (Vitro glucose reagent, Ortho Clinical Diagnostics, Rochester, NY, United States). β-HB was determined via a colorimetric β-HB dehydrogenase assay based on the reduction of the tetrazolium salt INT to a red-colored formazan dye with a color intensity directly proportional to β-HB concentration (Stanbio Chemistry Beta-Hydroxybutyrate LiquiColor® Assay, EKF Diagnostics USA, Boerne, TX, United States). All assays were run per manufacturer instructions.

### 2.2 Animals and sampling

Twenty-eight stingrays (15 males, 13 females) under managed care at the North Carolina Aquariums at Fort Fisher, Pine Knoll Shores, and Roanoke Island underwent routine annual preventative health exams and phlebotomy between September 2022 and October 2023. Four species were represented in the sample population: cownose rays (*Rhinoptera bonasus*, *n* = 6, min-max weight: 3.85–6.8 kg), Atlantic stingrays (*Hypanus sabina*, *n* = 10, min-max weight: 0.8–1.8 kg), southern stingrays (*Hypanus americanus*, *n* = 4, min-max weight: 5.4–9.2 kg), and yellow stingrays (*Urobatis jamaicensis*, *n* = 8, min-max weight: 0.6–1.65 kg). Stingrays were deemed clinically healthy for study inclusion via absence of abnormalities on physical exam (e.g., skin lesions, color changes, coelomic distension, etc.). All animals were fasted for 12–48 h and efficiently captured via a net from a holding or exhibit system in <5 min. Stingrays were anesthetized using 75–100 mg/L MS-222 buffered 1:1 by weight with sodium bicarbonate or marine buffer. After each stingray reached adequate anesthetic depth (approximately 5–10 min), blood was collected from a pectoral fin or mesopterygial vessel as previously described, using heparinized 22-gauge needles and 3 mL syringes (29). Needles and syringes were pre-heparinized before phlebotomy by aspirating a small volume of 1,000 U/mL sodium heparin and forcefully expelling it. Stingray handling and sampling was performed according to procedures approved by North Carolina State University’s Institutional Animal Care and Use Committee (IACUC #21–117).

### 2.3 Data collection

Immediately following phlebotomy, heparinized whole blood glucose and β-HB concentrations were measured using all three POC meters in a randomized order. Microhematocrit tubes were filled with heparinized whole blood, sealed with clay, and centrifuged for 5 min at 10,000 *g* (PowerSpin™ MH Centrifuge, UNICO, Dayton, NJ, United States). PCV and buffy coat (BC) were read using a hematocrit capillary tube reader (Veterinary Information Network®, Inc., Davis, CA, United States); total solids (TS) were determined via a handheld clinical refractometer (Jorgensen Laboratories, LLC, Loveland, CO, United States). The remainder of the heparinized whole blood sample was kept in a smaller cooler on ice until being centrifuged for 5 min at 3,000 *g* (Mini Centrifuge, Bio-Rad Laboratories, Inc., Hercules, CA, United States) within 1 h of sample collection. Heparinized plasma was separated from packed red blood cells (RBCs) using a disposable pipette, and plasma glucose and β-HB concentrations were measured using all three POC meters. The remaining plasma was placed in a 2-mL cryovial and shipped overnight to the glucose and β-HB reference laboratory (University of Miami, Miami, FL, United States). To measure POC meter precision (i.e., intra-assay variability or repeatability), heparinized whole blood glucose and β-HB concentrations were measured with each POC meter five times in rapid succession immediately following phlebotomy in a subset of two yellow stingrays. Heparinized plasma glucose and β-HB concentrations were measured with each POC meter five times in rapid succession immediately following centrifugation.

### 2.4 Statistical analyses

For glucose and β-HB concentrations below the lower limit of quantification (LLOQ) of the three POC meters and reference laboratory analyzer, imputation was performed by substituting half of the respective instrument’s LLOQ (e.g., if the LLOQ was 20 mg/dL, 10 mg/dL was inputted). To date, there is no widespread consensus on handling data below the LLOQ of an instrument (30). This study used imputation with half the LLOQ because it introduces less bias into the estimates than other single imputation approaches (30, 31). Summary statistics for glucose and β-HB were compiled for the three POC meters and reference laboratory analyzer. Using reference laboratory results, summary statistics of glucose and β-HB concentrations were also compiled by stingray species.

The diagnostic agreement (i.e., accuracy) between the three POC meters and reference laboratory results was assessed by calculating the observed total error (TE_obs_) and percent relative error, as well as using Bland–Altman methods (32) and Passing-Bablok regression (33, 34) for both glucose and β-HB using whole blood and plasma. The precision (i.e., intra-assay variability or repeatability) of the three POC meters for both glucose and β-HB using whole blood and plasma was assessed by calculating the percent coefficient of variation (CV) using five replicates with Equation 1:


(1) $$CV\;\left(\%\right)=\frac{\mathrm{Standard}\;\mathrm{Deviatio}n_{POC}}{Mean_{POC}}\times 100$$

Percent bias and TE_obs_ of the three POC meters for both glucose and β-HB using whole blood and plasma were calculated with Equations 2 and 3, respectively:


(2) $$\mathrm{Bias}\;\left(\%\right)=\left|\frac{Mean_{\mathrm{Reference}}-Mean_{POC}}{Mean_{\mathrm{Reference}}}\right|\times 100$$

(3) $$TE_{obs}\;\left(\%\right)=2\times CV\;\left(\%\right)+\mathrm{Bias}\;\left(\%\right)$$

The percent relative error between each POC meter result and the reference laboratory result was calculated with Equation 4 for glucose and β-HB:


(4) $$\mathrm{Relative}\;\mathrm{Error}\;\left(\%\right)=\left|\frac{\mathrm{Resul}t_{POC}-\mathrm{Resul}t_{\mathrm{Reference}}}{\mathrm{Resul}t_{\mathrm{Reference}}}\right|\times 100$$

The proportions of results with a percent relative error < 12, 15, and 20% were calculated for each POC meter for both glucose and β-HB using whole blood and plasma and compared to the US Food and Drug Administration relative error guidelines for human glucometers as previously described (26). For Bland–Altman plots and statistics, bias was calculated by subtracting the reference laboratory result from the POC meter result (32). The mean bias and the limits of agreement (LoAs) were then calculated for each POC meter for both glucose and β-HB using whole blood and plasma (32); LoAs were calculated with Equation 5:


(5) $$LoA=Mean_{\mathrm{Difference}}\pm \left(1.96\times \mathrm{Standard}\;\mathrm{Deviatio}n_{\mathrm{Difference}}\right)$$

Bias was considered statistically significant if the mean bias’s 95% confidence interval (95% CI) did not include 0 (32). For Passing-Bablok regression, constant bias was present if the *y*-intercept’s 95% CI did not include 0, whereas proportional bias was present if the slope’s 95% CI did not include 1 (33, 34).

To explore the effect of multiple variables on the difference between the POC meters and the reference laboratory analyzer, linear mixed-effect models were constructed for each POC meter for both glucose and β-HB. The difference between the POC meter and reference laboratory for either glucose and β-HB was included as the dependent variable; sex (male or female), weight, PCV, BC, and matrix (plasma or whole blood) were included as fixed effects; and stingray species and individual stingrays were included as random effects.

Data were collated in a standard spreadsheet software spreadsheet (Microsoft Excel, Microsoft Corporation, Redmond, WA, United States). All analyses were performed in the free statistical software R (v4.3.1, R Foundation for Statistical Computing, Vienna, Austria). *p* values <0.05 were considered significant. Data organization and summary statistics were performed using the dplyr (v1.1.2) (35) and psych (v2.3.6) (36) packages and their dependents. Bland–Altman plots and statistics were performed using the blandr (v0.5.3) (36) and ggplot2 (v3.4.3) (37) packages and their dependents. Passing-Bablok regression analyses were performed using the mcr package (v1.3.2) (38) and its dependents. Linear mixed-effect models were constructed using the lmerTest (v3.1.3) (39) package.

## 3 Results

Plasma glucose and β-HB concentrations, as determined by the reference laboratory, ranged from <20–63 mg/dL (median: 35 mg/dL) and 0.05–5.38 mmol/L (median: 0.22 mmol/L, Table 1), respectively. PCV, BC, and TS for all stingrays ranged from 11–32% (median: 24%), 0–4% (median: 0%), and 4.4–8.1 g/dL (median: 7.0 g/dL). Glucose concentration ranges as determined by the reference laboratory were similar across species: < 20–53 mg/dL (median: 26 mg/dL) for Atlantic stingrays, 32–59 mg/dL (median: 48 mg/dL) for cownose rays, 23–39 mg/dL (median: 36 mg/dL) for southern stingrays, and < 20–63 mg/dL (median: 30 mg/dL) for yellow stingrays. Similarly, β-HB concentration ranges as determined by the reference laboratory were similar across species: 0.1–0.4 mmol/L (median: 0.22 mmol/L) for Atlantic stingrays, 0.08–0.69 mmol/L (median: 0.31 mmol/L) for cownose rays, 0.14–0.28 mmol/L (median: 0.23 mmol/L) for southern stingrays, and 0.05–5.38 mmol/L (median: 0.22 mmol/L) for yellow stingrays with the maximum of the yellow stingray range being skewed by one individual (5.38 mmol/L). With the one apparent yellow stingray outlier removed, the β-HB concentration range was 0.05–0.52 mmol/L for conspecifics included in this study.

**Table 1:** Summary statistics of whole blood (WB) and plasma (P) glucose and β-hydroxybutyrate (β-HB) concentrations in stingrays determined by three commercial point-of-care glucose and β-HB meters and reference laboratory methods.

Analyte	Analyzer	Matrix	*n*	Mean	SD	Median	Min	Max
Glucose (mg/dL)	BHBCheck	WB	22	79	21	79	16	119
		P	22	99	30	99	56	154
	Precision Xtra	WB	28	35	20	39	< 20	71
		P	28	44	22	45	< 20	88
	Nova Vet™	WB	28	16	11	< 20	< 20	46
		P	28	20	15	< 20	< 20	54
	Reference Lab	P	28	34	15	35	< 20	63
β-HB (mmol/L)	BHBCheck	WB	28	0.7	0.8	0.6	0.2	4.4
		P	28	0.6	0.9	0.4	0.1	5.3
	Precision Xtra	WB	28	0.7	0.9	0.6	0.1	5.2
		P	28	0.5	0.8	0.3	0	4.3
	Nova Vet™	WB	28	0.4	0.5	0.3	< 0.1	2.9
		P	28	0.6	0.5	0.5	0.2	3.3
	Reference Lab	P	28	0.44	0.98	0.22	0.05	5.38

SD, Standard deviation; Min, Minimum; and Max, Maximum.

The CV or imprecision of the three POC meters for glucose was less when using plasma (maximum: 0–4.9%) than whole blood (maximum: 11%); similarly, the CV of the three POC meters for β-HB was less when using plasma (maximum: 0%) than whole blood (maximum: 12.5%). The CV was 0% for all three POC meters when measuring β-HB using plasma (Table 2). All three POC meters had less TE_obs_ for both whole blood and plasma when measuring β-HB than glucose (Table 2). The proportions of results with percent relative error < 12, 15, and 20% were tabulated for each POC meter for glucose and β-HB using whole blood and plasma (Table 2).

**Table 2:** Percent coefficient of variation (CV), bias, and observed total error (TE_obs_) for whole blood (WB) and plasma (P) glucose and β-hydroxybutyrate (β-HB) concentrations in stingrays determined by three commercial point-of-care glucose and β-HB meters.

Analyte	Analyzer	Matrix	*n*	CV	Bias	TE_obs_	< 12% RE	< 15% RE	< 20% RE
Glucose (%)	BHBCheck	WB	22	2.4	3188.2	3,193	0	0	0
		P	22	2.7	3127.4	3132.8	0	0	0
	Precision Xtra	WB	28	11	3316.8	3338.8	46.4	64.3	71.4
		P	28	4.9	3289.1	3298.9	21.4	21.4	35.7
	Nova Vet™	WB	28	0	3371.4	3371.4	17.9	17.9	21.4
		P	28	0	3359.1	3359.1	21.4	25	32.1
β-HB (%)	BHBCheck	WB	28	12.4	103	127.8	3.6	3.6	10.7
		P	28	0	88.4	88.4	10.7	10.7	10.7
	Precision Xtra	WB	28	0	103	103	14.3	14.3	14.3
		P	28	0	65.8	65.8	10.7	10.7	21.4
	Nova Vet™	WB	28	0	37.9	37.9	10.7	10.7	21.4
		P	28	0	80.3	80.3	10.7	14.3	21.4

The American Society for Veterinary Clinical Pathology guidelines advise TE_obs_ be <20% for point-of-care measurements within and above the reference interval. The percentages of measurements with percent relative error (RE) < 12, 15, and 20% are reported for each point-of-care meter, analyte, and matrix. The US Food and Drug Administration requires 95 and 98% of measurements to be within 12 and 15% of reference concentrations for glucometers intended for professional human healthcare use and 96 and 100% of measurements to be within 15 and 20% of reference concentrations for glucometers intended for over-the-counter use in human patients.

**Figure 1:**
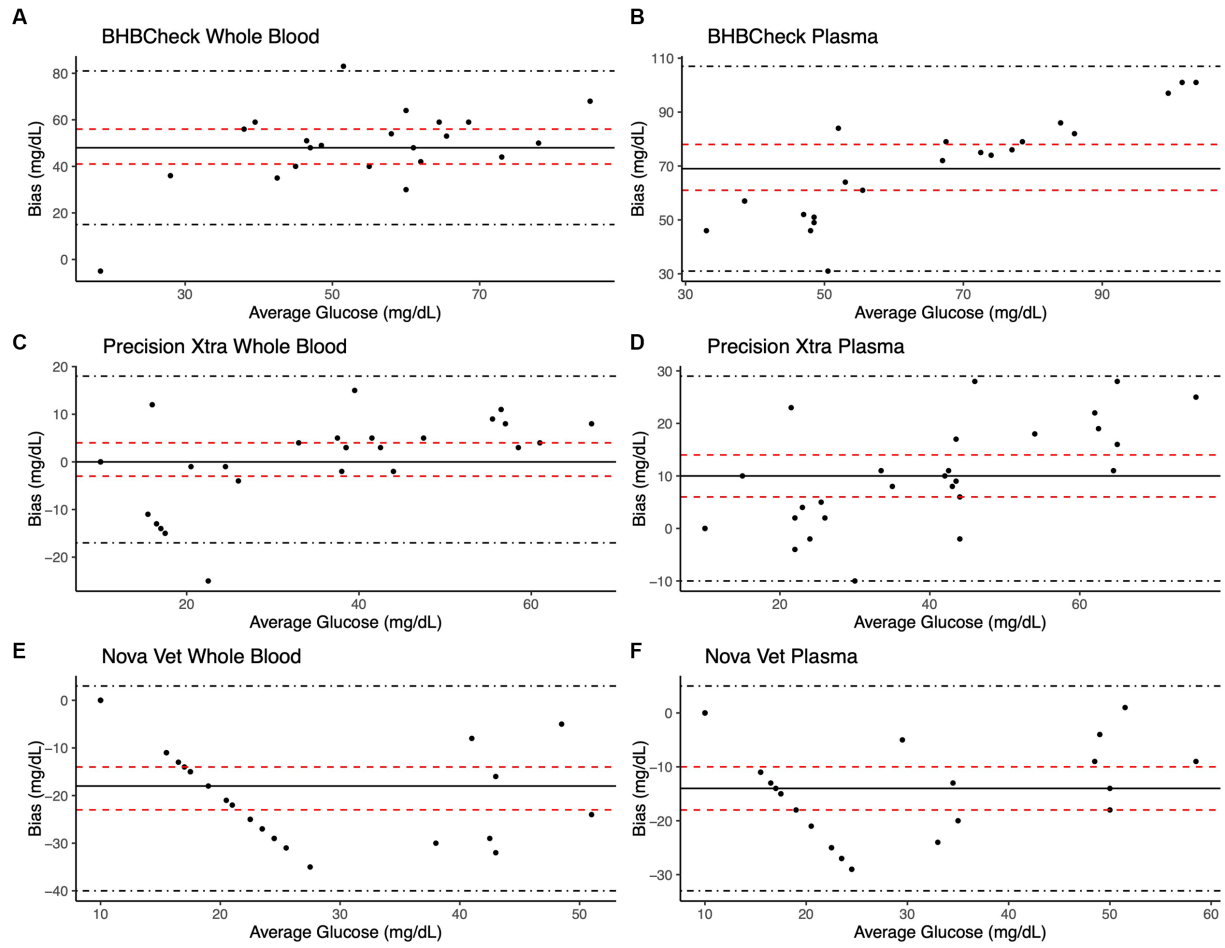
Bland–Altman plots of whole blood **(A, C, E)** and plasma **(B, D, F)** glucose concentrations in stingrays determined by three commercial point-of-care meters compared to reference laboratory methods. Black dots represent individual stingrays. The mean difference (Bias, solid black line) and the limits of agreement (LoA, black dot-dashed lines)—defined as the mean difference ± 1.96 times the standard deviation of the differences—were calculated for each meter. Bias was considered statistically significant if the 95% confidence interval (red dashed lines) mean difference did not include 0.

**Figure 2:**
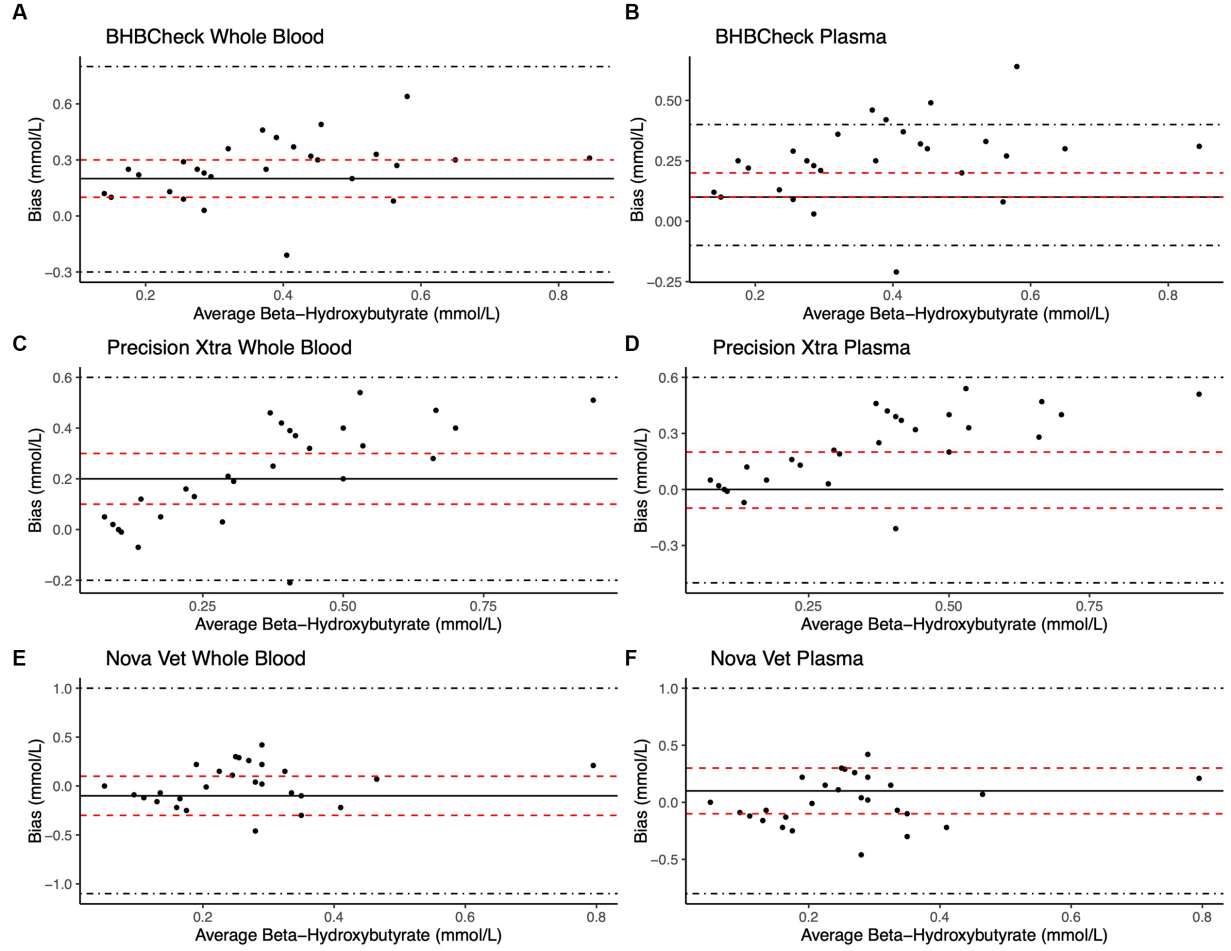
Bland–Altman plots of whole blood **(A, C, E)** and plasma **(B, D, F)** β-hydroxybutyrate (β-HB) concentrations in stingrays determined by three commercial point-of-care meters compared to reference laboratory methods. Black dots represent individual stingrays. The mean difference (Bias, solid black line) and the limits of agreement (LoA, black dot-dashed lines)—defined as the mean difference ± 1.96 times, the standard deviation of the differences—were calculated for each meter. Bias was considered statistically significant if the 95% confidence interval (red dashed lines) of the mean difference did not include 0. To avoid skewing the Bland–Altman plots, a single yellow stingray outlier was removed (β-HB = 5.38 mmol/L via the reference laboratory method).

Bland–Altman methods found the BHBCheck meter had significant positive bias (i.e., overestimated) for both glucose and β-HB regardless of the matrix (Table 3; Figures 1, 2). The Precision Xtra meter had a significant positive bias for glucose using plasma and β-HB using whole blood (Table 3; Figures 1, 2). The Nova Vet™ had significant negative bias (i.e., underestimated) for glucose regardless of the matrix (Table 3; Figures 1, 2). For glucose, the mean bias ranged from −18 to 48 mg/dL using whole blood and from −14 to 69 mg/dL using plasma across the three POC meters (Table 3). For β-HB, the mean bias ranged from −0.1 to 0.2 mmol/L using whole blood and from 0 to 0.1 mmol/L using plasma across the three POC meters (Table 3).

**Table 3:** Bland–Altman statistics and Passing-Bablok regression results for whole blood (WB) and plasma (P) glucose and β-hydroxybutyrate (β-HB) concentrations in stingrays determined by three commercial point-of-care glucose and β-HB meters and reference laboratory methods.

			Bland–Altman statistics			Passing-Bablok regression	
Analyte	Analyzer	Matrix	Bias	Lower LoA	Upper LoA	Intercept	Slope
Glucose (mg/dL)	BHBCheck	WB	48 (41, 56)^*^	15 (2, 28)	81 (68, 94)	37 (3, 55)^*^	1.4 (0.9, 2.4)
		P	69 (61, 78)^*^	31 (17, 46)	107 (92, 121)	29 (8, 46)^*^	2.3 (1.9, 2.8)^*^
	Precision Xtra	WB	0 (−3, 4)	−17 (−23, −11)	18 (12, 24)	−6 (−21, −1)^*^	1.3 (1.1, 1.6)^*^
		P	10 (6, 14)^*^	−10 (−17, −3)	29 (23, 36)	−5 (−15, 1)	1.4 (1.3, 1.7)^*^
	Nova Vet™	WB	−18 (−23, −14)^*^	−40 (−47, −32)	3 (−4, 10)	10 (−7, 10)	0 (0, 0.6)^*^
		P	−14 (−18, −10)^*^	−33 (−39, −27)	5 (−2, 11)	−5 (−20, 10)	0.7 (0, 1.1)
β-HB (mmol/L)	BHBCheck	WB	0.2 (0.1, 0.3)^*^	−0.3 (−0.5, −0.1)	0.8 (0.6, 1)	0.2 (0, 0.3)	1.3 (0.9, 2.5)
		P	0.1 (0.1, 0.2)^*^	−0.1 (−0.1, 0)	0.4 (0.3, 0.4)	0.1 (0.1, 0.2)^*^	1.1 (1, 1.4)
	Precision Xtra	WB	0.2 (0.1, 0.3)^*^	−0.2 (−0.3, −0.1)	0.6 (0.5, 0.8)	0 (−0.2, 0.1)	2 (1.5, 2.7)^*^
		P	0 (−0.1, 0.2)	−0.5 (−0.7, −0.3)	0.6 (0.4, 0.8)	−0.1 (−0.2, 0)	1.7 (1.3, 2.2)^*^
	Nova Vet™	WB	−0.1 (−0.3, 0.1)	−1.1 (−1.4, −0.7)	1 (0.6, 1.3)	0 (−0.2, 0.7)	1.1 (−2.2, 1.8)
		P	0.1 (−0.1, 0.3)	−0.8 (−1.1, −0.5)	1 (0.7, 1.3)	0.5 (0.3, 0.6)^*^	0 (−0.4, 0.6)^*^

The mean difference (Bias) and the limits of agreement (LoA)—defined as the mean difference ± 1.96 times the standard deviation of the differences—were calculated for each meter. Bias was considered statistically significant (^*^) if the mean difference’s 95% confidence interval (95% CI) did not include 0. For Passing-Bablok regression, constant bias was present (^*^) if the 95% CI for the *y*-intercept did not include 0, whereas proportional bias was present (^*^) if the 95% CI for the slope did not include 1.

**Figure 3:**
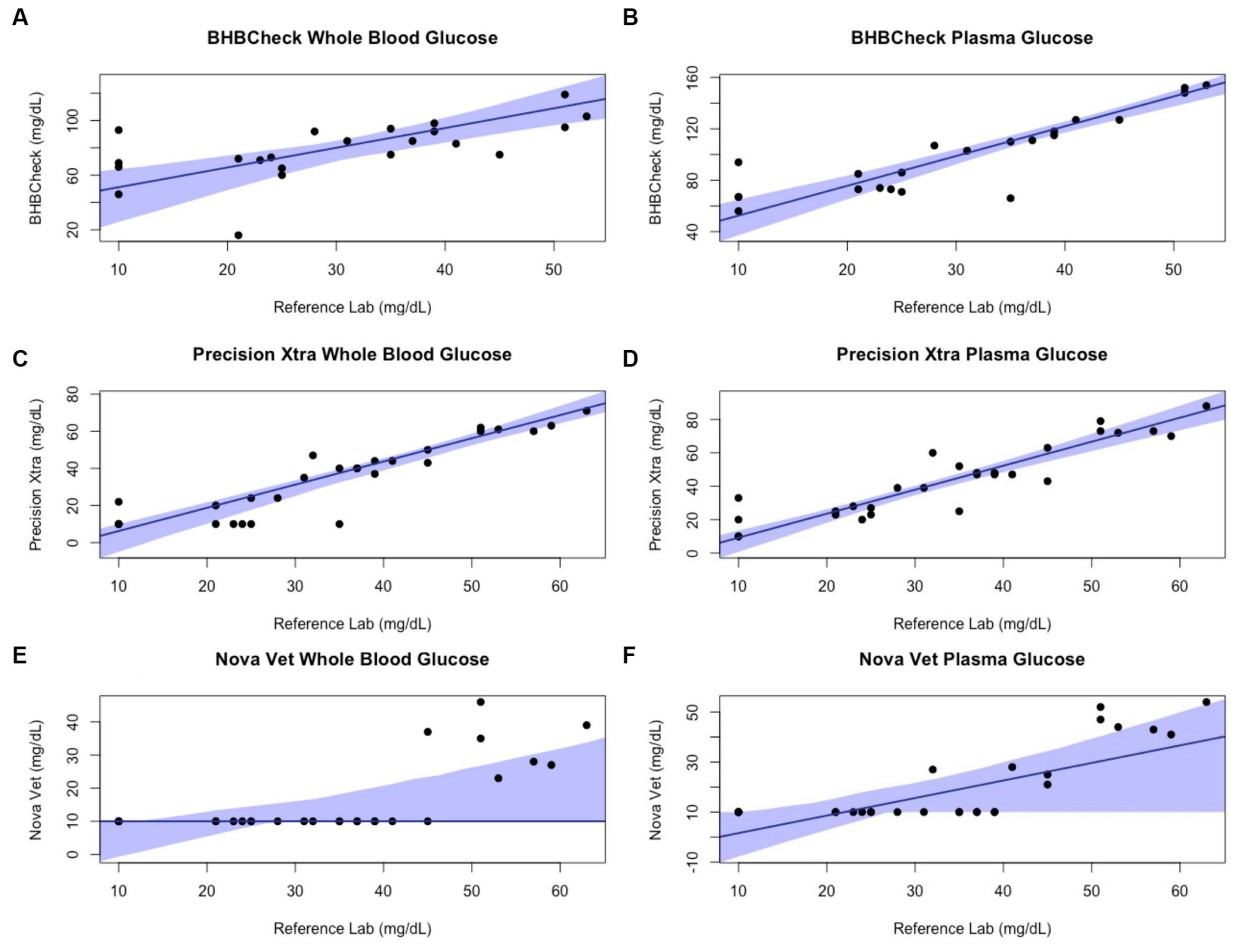
Passing-Bablok plots of whole blood and plasma glucose concentrations in stingrays determined by three commercial point-of-care meters compared to reference laboratory methods. Black dots represent individual stingrays. The solid blue line and associated shading represent the estimated regression line and its 95% confidence interval. For Passing-Bablok regression, constant bias was present if the 95% CI for the *y*-intercept did not include 0, whereas proportional bias was present if the 95% CI for the slope did not include 1.

**Figure 4:**
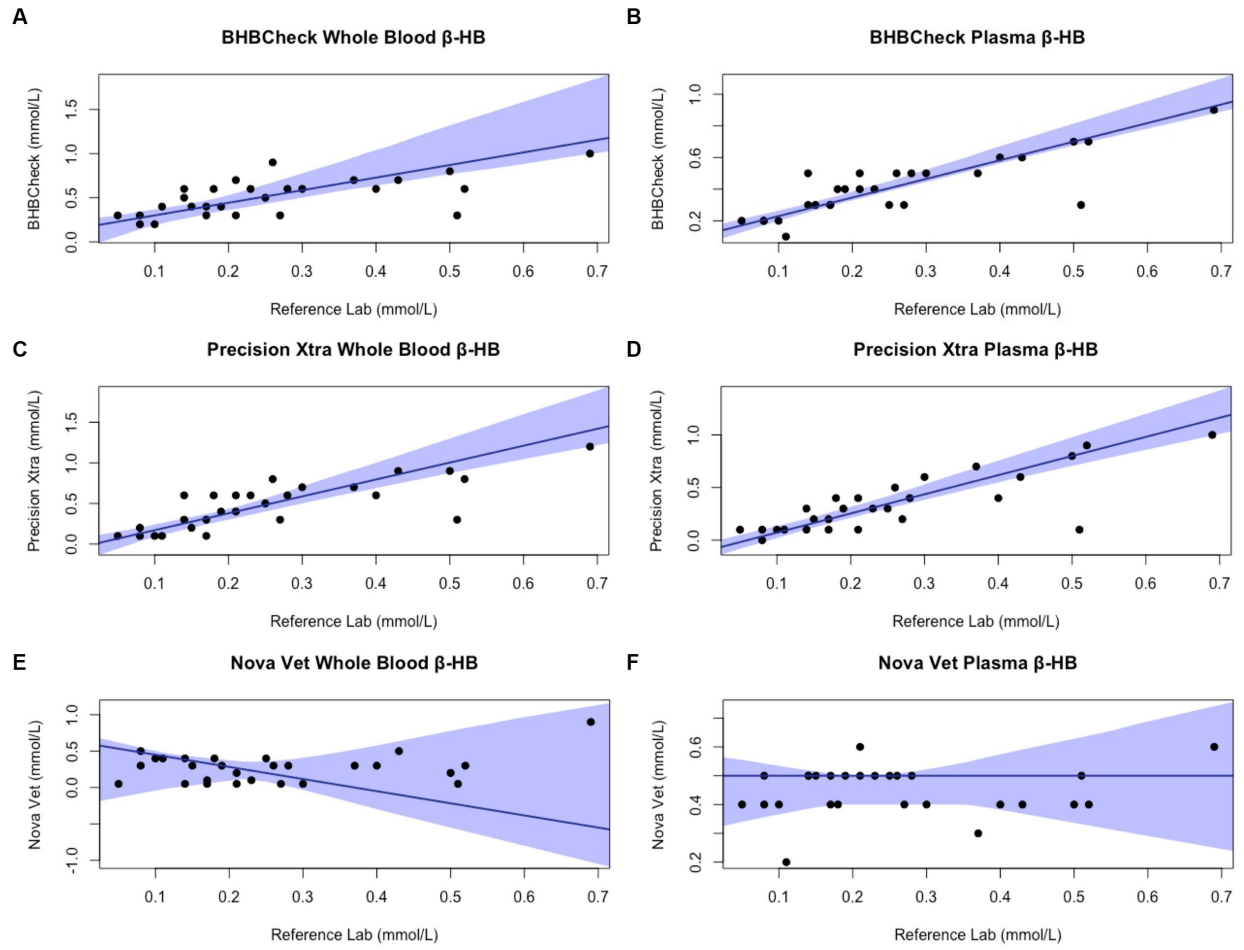
Passing-Bablok plots of whole blood and plasma β-hydroxybutyrate (β-HB) concentrations in stingrays determined by three commercial point-of-care meters compared to reference laboratory methods. Black dots represent individual stingrays. The solid blue line and associated shading represent the estimated regression line and its 95% confidence interval. For Passing-Bablok regression, constant bias was present if the 95% CI for the *y*-intercept did not include 0, whereas proportional bias was present if the 95% CI for the slope did not include 1. To avoid skewing the Passing-Bablok plots, a single yellow stingray outlier was removed (β-HB = 5.38 mmol/L via the reference laboratory method).

Passing-Bablok regression analyses found that the BHBCheck meter had a significant constant bias for glucose regardless of matrix and β-HB using plasma and a significant proportional bias for glucose using plasma (Table 3; Figures 3, 4). The Precision Xtra meter had a significant constant bias for glucose using whole blood and β-HB using plasma, as well as a significant proportional bias for both glucose and β-HB regardless of the matrix (Table 3; Figures 3, 4). The Nova Vet™ meter had a significant constant bias for β-HB using plasma and a significant proportional bias for glucose using whole blood and β-HB using plasma (Table 3; Figures 3, 4).

Linear mixed-effect models found sex, weight, PCV, and BC had no significant effect on differences between the individual POC meters and the reference laboratory for glucose or β-HB. Matrix had no significant effect on differences between the BHBCheck or Nova Vet™ meters and the reference laboratory methods for glucose or β-HB. However, matrix did have a significant effect on β-HB, but not glucose, for the Precision Xtra meter: differences in β-HB concentrations were significantly lower using whole blood than plasma (matrix estimate ± standard error: −2.3 ± 0.71 mmol/L, *p* value = 0.002).

## 4 Discussion

This study evaluated the diagnostic agreement (i.e., accuracy) between three commercial POC glucose and β-HB meters to reference laboratory methods using whole blood and plasma from four stingray species commonly found in managed care environments. Plasma glucose concentrations determined by a reference laboratory method were similar to previous reports in cownose rays and southern stingrays (40–42); likewise, β-HB concentrations determined by a reference laboratory method fell within previously reported ranges in elasmobranchs (4). Of the three POC meters evaluated, the Precision Xtra—a human-licensed glucometer—showed the greatest agreement with the reference laboratory methods when measuring glucose from whole blood (mean bias: 0 mg/dL) and measuring β-HB from plasma (mean bias: 0.1 mmol/L) in stingrays. Stingray sex, weight, PCV, and BC did not significantly affect differences between the POC meters and the reference laboratory results. All three POC meters had better precision (i.e., intra-assay variability or repeatability) when using plasma than whole blood to measure glucose. Regarding the study’s hypothesis, the mean bias and CV for plasma β-HB concentrations was relatively small (min-max: 0–0.1 mmol/L and 0%, respectively) across all three POC meters, suggesting POC β-HB measurement may have sufficient agreement with reference laboratory methods and precision for clinical and field research purposes.

The American Society for Veterinary Clinical Pathology (ASVCP) has developed guidelines for quality assurance and control of POC glucose measurement in light of the rising number of commercially available POC glucometers (43). Point-of-care meter error can be categorized as pre-analytical, analytical, and post-analytical (43). Pre-analytical error was minimized in the present study by adequate observer training and using similar sampling techniques and venipuncture sites. However, species- or individual-specific matrix properties (e.g., blood viscosity, rheology, mean corpuscular volume, etc.) may have contributed to error between the three POC meters and the reference laboratory analyzer, particularly for glucose concentrations. The distribution of glucose between RBCs and plasma is an important matrix-related variable across species (43). Glucose is distributed equally between RBCs and plasma in humans, whereas in canine, feline, rabbit, and avian patients, glucose in circulation is found predominantly in plasma (43). To the authors’ knowledge, the distribution of glucose between RBCs and plasma in the four stingray species sampled is unknown and remains a potential avenue of future physiologic research. Analytical error was minimized during data collection by using test strips before they expired, routine calibration using manufacturer-provided control materials, and not operating the POC meters under extreme environmental conditions. Post-analytical error was avoided by using consistent units of measurement (mg/dL for glucose, mmol/L for β-HB) and recording results immediately after being displayed by the POC meters.

In human and veterinary patients, anemia or hemoconcentration can falsely increase or decrease POC meter glucose or β-HB measurements (15, 18, 21, 43, 44). In stingrays, linear mixed-effect models found PCV had no significant effect on differences between the three POC meters and the reference laboratory analyzer, despite a subset of sampled stingrays with PCV values lower than the recommended ranges for the meter. Possible reasons PCV influences POC meter glucose or β-HB measurements include built-in conversion factors or increased diffusion rate in test strips with lower PCV (43). This study did not assess whether analytical interference occurred due to other blood gas or biochemical parameters such as pH, pO_2_, cholesterol, bilirubin, or biliverdin (43). All sampled stingrays were anesthetized with MS-222 to ensure safe and efficient handling, which could have resulted in analytical interference secondary to perianesthetic physiologic changes (e.g., acid–base status, body temperature, ventilation, vasoconstriction, etc.). Although veterinary studies are lacking, POC glucometers are less accurate intraoperatively in human patients under anesthesia (45, 46).

The ASVCP guidelines advise TE_obs_ for laboratory instruments, including POC glucose and β-HB meters, to be <10% for values below the reference interval or < 20% for values within and above the reference interval (43, 47). All three POC meters for both glucose and β-HB using whole blood and plasma had TE_obs_ > 20%, indicative of undesirable analytical performance. Furthermore, the US Food and Drug Administration requires 95 and 98% of measurements to be within 12 and 15% of reference concentrations for POC glucometers intended for professional healthcare use and 96 and 100% of measurements to be within 15 and 20% of reference concentrations for POC glucometers intended for over-the-counter use (26). All three POC meters evaluated did not meet US Food and Drug Administration relative error guidelines for human glucometers when measuring whole blood or plasma glucose concentrations in stingrays (27). To the authors’ knowledge, neither the US Food and Drug Administration nor ASVCP has produced guidelines for POC β-HB meters despite their frequent use in monitoring diabetes mellitus in humans and domestic small animals.

Regardless of specific statistical analyses employed to compare new and established diagnostic methods, it is essential to appraise the clinical relevance of their analytic agreement. Clarke or Parkes Error Grid Analysis has historically been used in human and veterinary POC glucometer research to understand better potential clinical outcomes associated with POC meter accuracy (48, 49). This study implemented neither method due to the wide range of glucose and β-HB concentrations observed in elasmobranchs and little consensus on pathophysiologic thresholds for either analyte (e.g., hypoglycemic, ketonemia). However, the BHBCheck meter could potentially lead to highly inaccurate glucose interpretations due to the magnitude of its mean bias using whole blood (48 mg/dL) or plasma (69 mg/dL) and the narrow range of glucose concentrations observed by the reference laboratory (< 20–63 mg/dL). Passing-Bablok regression analyses found that all three POC meters mainly exhibited significant positive proportional bias (i.e., 95% CI > 1) measuring glucose and β-HB, signifying greater error at higher concentrations. Consequently, future research should assess agreement with a reference laboratory under potential pathophysiologic states or physiologic extremes (e.g., hyperglycemia secondary to long-line capture, ketonemia due to prolonged fasting, etc.).

Limitations of the present study include limited sample sizes per species (*n* < 10) and variable fasting durations before sampling, given differences in husbandry routines at the separate facilities. While imputation by substituting values below the LLOQ with a constant was employed in the present study, multiple imputation, maximum likelihood estimation, or kernel density estimation may be better alternative approaches. Although variation in venipuncture sites can influence PCV in sharks (50) and downstream biochemical analyses in other taxa, no statistically significant differences between venipuncture sites were found in southern stingrays (51). Although plasma was separated from RBCs promptly via centrifugation, glucose and β-HB concentrations could have decreased slightly between venipuncture and processing due to metabolism by cellular constituents.

Rapid, reliable detection of hyperglycemia due to physiologic stress could help veterinarians and researchers working with elasmobranchs more objectively monitor their welfare during capture, handling, and transport. Similarly, accurate POC measurement of β-HB could provide almost instant, cost-effective insight into the metabolic state of the elasmobranch patient vs. the turnaround time and expense of sending a sample to a reference laboratory. Further research should investigate POC meter clinical utility under different metabolic states in elasmobranchs. Using POC meters to measure glucose and β-HB concentrations in stingrays may be possible when reference methods are inaccessible. β-HB measurement using plasma will likely have sufficient accuracy and precision for clinical and field research purposes for the three POC meters evaluated in this study, especially the human-licensed glucometer, Precision Xtra.

## Data availability statement

The raw data supporting the conclusions of this article will be made available by the authors, without undue reservation.

## Ethics statement

The animal study was approved by North Carolina State University Institutional Animal Care and Use Committee. The study was conducted in accordance with the local legislation and institutional requirements.

## Author contributions

ND: Conceptualization, Investigation, Data Curation, Formal analysis, Writing – original draft, Writing – review & editing. CC: Writing – review & editing. LW: Writing – review & editing. EC: Writing – review & editing.
